# Boils

**Published:** 1844-01-13

**Authors:** 


### BOILS.

You cannot disperse them, even if you ought; you may try, therefore, to bring the boil forward by steaming ; but you had better cover it with plaster, and attend to its source, and prevent others, by attention to the stomach, by an emetic and alterative pill, and bitter infusion with alkaline solutions. When it looks ill, and exhibits a mass of corrupted cellular membrane, it should be dressed with digestive ointment and poulticed. To correct the disposition to them, after considering the state of the intestinal canal, give antimonials, and order the warm bath.—*Sir Charles Bell, from London Lancet.*

